# Shortening door-to-puncture time and improving patient outcome with workflow optimization in patients with acute ischemic stroke associated with large vessel occlusion

**DOI:** 10.1186/s12873-022-00692-8

**Published:** 2022-07-26

**Authors:** Shuiquan Yang, Weiping Yao, James E. Siegler, Mohammad Mofatteh, Jack Wellington, Jiale Wu, Wenjun Liang, Gan Chen, Zhou Huang, Rongshen Yang, Juanmei Chen, Yajie Yang, Zhaohui Hu, Yimin Chen

**Affiliations:** 1 Department of Neurology and Advanced National Stroke Center, Foshan Sanshui District People’s Hospital, No. 16, Guanghaidadaoxi, Sanshui District, Foshan, 528100 Guangdong Province China; 2 Dean Office and Advanced National Stroke Center, Foshan Sanshui District People’s Hospital, Foshan, Guangdong Province China; 3 Cooper Neurological Institute, Cooper University Hospital, Camden, NJ USA; 4 School of Medicine, Dentistry and Biomedical Sciences, Queen’s University Belfast, Belfast, UK; 5 School of Medicine, Cardiff University, Wales, UK; 6 Foshan Sanshui District People’s Hospital, Foshan, Guangdong Province China; 7 School of Medicine, Shaoguan University, Shaoguan, Guangdong Province China; 8 Department of Radiology, Foshan Sanshui District People’s Hospital, Foshan, Guangdong Province China; 9 The Second Clinical College, Guangzhou Medical University, Guangzhou, Guangdong Province China; 10 The First School of Clinical Medicine, Southern Medical University, Guangzhou, Guangdong Province China; 11 Medical Department and Advanced National Stroke Center, Foshan Sanshui District People’s Hospital, Foshan, Guangdong Province China

**Keywords:** Door-to-puncture time, Door-to-recanalization time, Puncture-to-recanalization time, Ischemic stroke, Endovascular therapy, Workflow optimization

## Abstract

**Objective:**

We aimed to evaluate door-to-puncture time (DPT) and door-to-recanalization time (DRT) without directing healthcare by neuro-interventionalist support in the emergency department (ED) by workflow optimization and improving patients’ outcomes.

**Methods:**

Records of 98 consecutive ischemic stroke patients who had undergone endovascular therapy (EVT) between 2018 to 2021 were retrospectively reviewed in a single-center study. Patients were divided into three groups: pre-intervention (2018–2019), interim-intervention (2020), and post-intervention (January 1^st^ 2021 to August 16^th^, 2021). We compared door-to-puncture time, door-to-recanalization time (DRT), puncture-to-recanalization time (PRT), last known normal time to-puncture time (LKNPT), and patient outcomes (measured by 3 months modified Rankin Scale) between three groups using descriptive statistics.

**Results:**

Our findings indicate that process optimization measures could shorten DPT, DRT, PRT, and LKNPT. Median LKNPT was shortened by 70 min from 325 to 255 min(*P* < 0.05), and DPT was shortened by 119 min from 237 to 118 min. DRT shortened by 132 min from 338 to 206 min, and PRT shortened by 33 min from 92 to 59 min from the pre-intervention to post-intervention groups (all *P* < 0.05). Only 21.4% of patients had a favorable outcome in the pre-intervention group as compared to 55.6% in the interventional group (*P*= 0.026).

**Conclusion:**

This study demonstrated that multidisciplinary cooperation was associated with shortened DPT, DRT, PRT, and LKNPT despite challenges posed to the healthcare system such as the COVID-19 pandemic. These practice paradigms may be transported to other stroke centers and healthcare providers to improve endovascular time metrics and patient outcomes.

## Introduction

Stroke affects one-fifth of the world’s population and is the leading cause of mortality in China [1]. Endovascular thrombectomy has been proven to reduce disability in ischemic stroke patients with large vessel occlusion when performed within 6 h, or in selected patients up to 24 h post-stroke onset [2]. More favorable patient outcomes are observed when shorter delays in pre-hospital care and cumulative time from symptom recognition to treatment [3, 4]. Endovascular treatment has also been associated with a lower risk of complications, including symptomatic intracranial hemorrhage (sICH), achieving discharge independent walking, and lower in-hospital death or hospice discharge when patients are treated soon after ictus [3, 4]. Current guidelines strongly recommend providers effectively shorten intraarterial therapy time for patients with ischemic stroke to improve patient outcomes and explore process improvement initiatives to optimize patient throughput [5]. One study has demonstrated that the direct involvement of neuro-interventionalists in the emergency department (ED) could shorten the door-to-puncture time (DPT) from 167.2 ± 54.3 min to 135.2 ± 50.0 min (*P* = 0.040) [6]. Another study showed that multidisciplinary cooperation with regular training and debriefing might also shorten the door-to-needle time (DNT) even during the COVID-19 pandemic [7]. Our Foshan Sanshui District People’s hospital is the only comprehensive tertiary hospital and national stroke center that serves more than 0.8 million people, providing intravenous (IV) thrombolysis and endovascular therapy for acute ischemic stroke patients. Due to staffing availability, our neuro-interventionalists do not respond to the ED for stroke codes. With that in mind, we aimed to shorten the DPT and door-to-recanalization time (DRT) without the involvement of neuro-interventionalist support in the ED through nursing and provider education, process optimization, and faster facilitation of transfer of patients between departments.

## Methods

### Design and setting

This study included the retrospective analysis of prospectively collected data from 98 consecutive ischemic stroke patients who underwent endovascular therapy from 2018 to 2021 in a single-center study in Foshan Sanshui District People’s Hospital in China. We compared time to interventions across three patient groups according to timing of intervention: pre-intervention (2018–2019; *n* = 14), interim-intervention (2020; *n* = 39), and post-intervention (January 1^st^ 2021 to August 16^th^, 2021; *n* = 45). Inclusion criteria were as follows: age ≥ 18 years old; admitting diagnosis of acute ischemic stroke due to an acute occlusion of the internal carotid artery, M1 or M2 segments of the middle cerebral artery, or basilar artery; stroke onset or last known well within 24 h of thrombectomy. The hospital institutional review board approved the study protocol Informed consent was waived due to the nature of a retrospective observational study.

### Data collection

For all patients included in this study, we recorded the following demographics and information: age, sex, past medical history of hypertension, atrial fibrillation (AF), diabetes mellitus (DM), chronic kidney disease (CKD), coronary heart disease (CAD), dyslipidemia, history of stroke, and smoking status. Neurologists measured and recorded the National Institute of Health Stroke Scale (NIHSS), Pre- endovascular therapy (EVT) Alberta Stroke Program Early CT Score (ASPECTS), initial premorbid modified Rankin Scale (mRS), Trial of ORG 10,172 in Acute Stroke Treatment (TOAST) stroke classification, and treatment with IV thrombolysis. DPT, DRT, puncture-to-recanalization time (PRT), and last known normal-to-puncture time (LKNPT) were collected. Three-month mRS scores were evaluated by routine follow-up.

### Interventions

Potential improvement points were identified in our hospital by multiple discussions and meetings with medical colleagues, the hospital chief and staff. Table 1 summarizes improvement measures implemented. Each measure was introduced and implemented during the interim-intervention period.

**Table 1:** A summary of improvement measures implemented with details provided for each measure

Measures	Details
Chief of hospital engagement	The chief of the hospital was engaged in the introduction process of the measures to facilitate improving stroke workflow
Pre-notification	A pre-notification system was established via referral hospital doctors to communicate a history of patients from the next of kin and assess thrombectomy treatment benefits and risks for suspected ischemic large vessel occlusion patients
Training	Multiple training sessions were provided for stroke and emergency nurses to promptly recognize stroke signs and symptoms
Priority	Suspected ischemic stroke patients were prioritized for triage by an emergency doctor
	CT was prioritized for suspected ischemic stroke patients
	CTA or MRA for suspected ischemic stroke large vessel occlusion patients within 24 h of onset was prioritized
	When CTA was performed, CTA images were reconstructed by radiologists in real-time to facilitate rapid imaging interpretation
	CT was primarily used for all patients, but MRI/MRA/CTP/MRP was prioritized for suspected ischemic stroke patients
	Neurointerventionalist availability for emergency procedures was prioritized for patients with intracranial occlusion
Reduce procedures	Implementation of a modified direct-to-Digital Subtraction Angiography approach, bypassing CTA for selected patients with a clinical suspicion of large vessel occlusion and lack of intracranial hemorrhage on initial CT
	More rapid acquisition of consent with support of other providers
Neuro-interventionists team cooperation	Cooperation of two experienced neuro-interventionists, with one discussing with patients’ family members to acquire consent for thrombectomy, and the other preparing patients for thrombectomy
Green light route	Medical department decision in the best interest of the patient to whether thrombectomy could be performed in critical or emergency situations if a patient family member could be contacted
	Surgery was provided without delays for hospital fees payment for all patients
Prepare in advance	Preparation of the medications and required devices for thrombectomy in advance by an interventional nurse once the notification is received
Feedback	Holding monthly stroke meetings to analyze the etiology of DPT-delayed cases by hospital chief and the ED staff, neurology, and radiology department staff
Reward	Rewarding participation of intervention center, ED staff, neurology, and radiology departments financially if DPT was performed less than or equal to 120 min and if patient outcomes were above satisfactory level
Public education	Increasing the awareness of the public about the signs and symptoms of acute stroke and thrombectomy by using local newspapers, television programs and the Internet platform by Regional Health Bureau and Media Department of the hospital

Abbreviations- *CT* Computerized tomography, *CTA* Computed tomography angiography, *CTP* Computed tomography perfusion, *DPT* Door-to-puncture time, *ED* Emergency department, *MRA* Magnetic resonance angiography, *MRP* Magnetic resonance perfusion

### Outcome measurements

The modified Treatment In Cerebral Infarction (mTICI) score was used to assess the recanalization rate [8]. Successful recanalization was defined as TICI 2b to 3. Modified Rankin scale (mRS) scores were determined by phone calls or in-person outpatient appointments and used to assess patient outcomes at 90 days, which was collected by a trained and dedicated stroke nurse navigator following the implementation period, as required for certification of a national stroke center [9]. The favorable outcome was defined as mRS 0–2 at 90 days. Symptomatic intracranial hemorrhage (sICH) was defined by The Heidelberg Bleeding Classification as a new intracranial hemorrhage associated with ≥ 4-point worsening in NIHSS, or ≥ 2-point worsening in a single NIHSS item—neither of which would be attributed to a process other than the hemorrhage [10].

### Statistical analyses

The non-parametric Mann–Whitney U test was performed using IBM SPSS version 23 (IBM-Armonk, NY) to analyze non-normally distributed continuous data, reported as medians along with the interquartile range (IQR). Normally distributed data are reported as means with corresponding standard deviations (SD) and compared using the student’s t-test. Results were considered statistically significant if the *P*-value was less than 0.05. No adjustments were made for multiple hypotheses testing. The results were reported using the STrengthening the Reporting of OBservational Studies in Epidemiology (STROBE) guidelines [11].

## Results

There were 98 patients evaluated during the study period who were included in the final analysis. There were no statistically significant differences regarding age, sex, cerebrovascular risk factors, mRS pre-treatment, pre-treatment ASPECTS, and IV thrombolysis of study participants between pre-intervention, interim-intervention, and post-intervention groups (Table 2). Admission NIHSS (IQR) of study participants between pre-intervention, interim-intervention and post-intervention groups were 19.0 (11.0, 21.0), 14.0 (11.0, 18.0), and 17.0 (14.0, 21.0) respectively (*P* = 0.026). There was a significant distribution in stroke mechanisms between the study periods based on TOAST definition (*P* = 0.028; Table 2).

**Table 2:** Clinical and imaging data for different phases of the study. P values are provided for each component

	Pre-intervention	Interim-intervention	Post-intervention	P
Number	14	39	45	
Age, mean ± SD	61.57	66.87	65.29	0.434
Male, n, %	11 (78.6%)	30 (76.9%)	31 (68.9%)	0.643
Hypertension, n, %	6 (42.9%)	24 (61.5%)	30 (66.7%)	0.679
AF, n, %	6 (42.9%)	13 (33.3%)	17 (37.8%)	0.802
DM, n, %	1 (7.1%)	6 (15.4%)	11 (24.4%)	0.284
CAD, n, %	4 (28.6%)	9 (23.1%)	9 (20.0%)	0.792
Previous Stroke, n, %	1 (7.1%)	10 (25.6%)	9 (20.0%)	0.336
Dyslipidemia, n, %,	1 (7.1%)	5 (12.8%)	9 (20.0%)	0.434
CKD, n, %	0 (0.0%)	3 (7.7%)	7 (15.6%)	0.193
Smoker, n, %	7 (50.0%)	12 (30.8%)	9 (20.0%)	0.088
mRS pre-treatment (IQR)	0.0 (0.0,0.0)	0.0 (0.0,0.0)	0.0 (0.0,0.0)	0.597
pre-treatment ASPECTS (IQR)	9.0 (8.0,9.0)	8.0 (8.0,9.0)	8.0 (7.5,9.0)	0.184
Admission NIHSS (IQR)	19.0 (11.0,21.0)	14.0 (11.0,18.0)	17.0 (14.0,21.0)	0.026
Vessels occlusion				
ICA, n, %	2 (14.3%)	8 (20.5%)	6 (13.3%)	0.697
M1, n, %	5 (35.7%)	16 (41.0%)	22 (48.9%)	
M2, n, %	0 (0.0%)	1 (2.6%)	0 (0.0%)	
Basilar artery, n, %	5 (35.7%)	8 (20.5%)	14 (31.1%)	
Tandem, n, %	2 (14.3%)	6 (15.4%)	3 (6.67%)	
TOAST type				
LAA, n, %	3 (21.4%)	22 (56.4%)	19 (42.2%)	0.028
CE, n, %	6 (42.9%)	15 (38.5%)	22 (51.2%)	
SVO, n, %	0 (0.0%)	1 (2.6%)	0 (0.0%)	
SOE, n, %	3 (21.4%)	0 (0.0%)	2 (4.4%)	
SUE, n, %	2 (14.3%)	1 (2.6%)	2 (4.4%)	
IV Thrombolysis, n, %	7 (50.0%)	18 (46.2%)	23 (51.1%)	0.942

Abbreviations – *AF* Atrial fibrillation, *DM* Diabetes mellitus, *CKD* Chronic kidney disease, *CAD* Coronary heart disease, *ICA* Internal carotid artery, *IV* Intravenous, *LAA* Large-artery atherosclerosis, *CE* Cardioembolism, *SOE* Stroke of undetermined etiology, *SUE* Stroke of undetermined etiology, *SVO* Small vessel occlusion

**Fig. 1:**
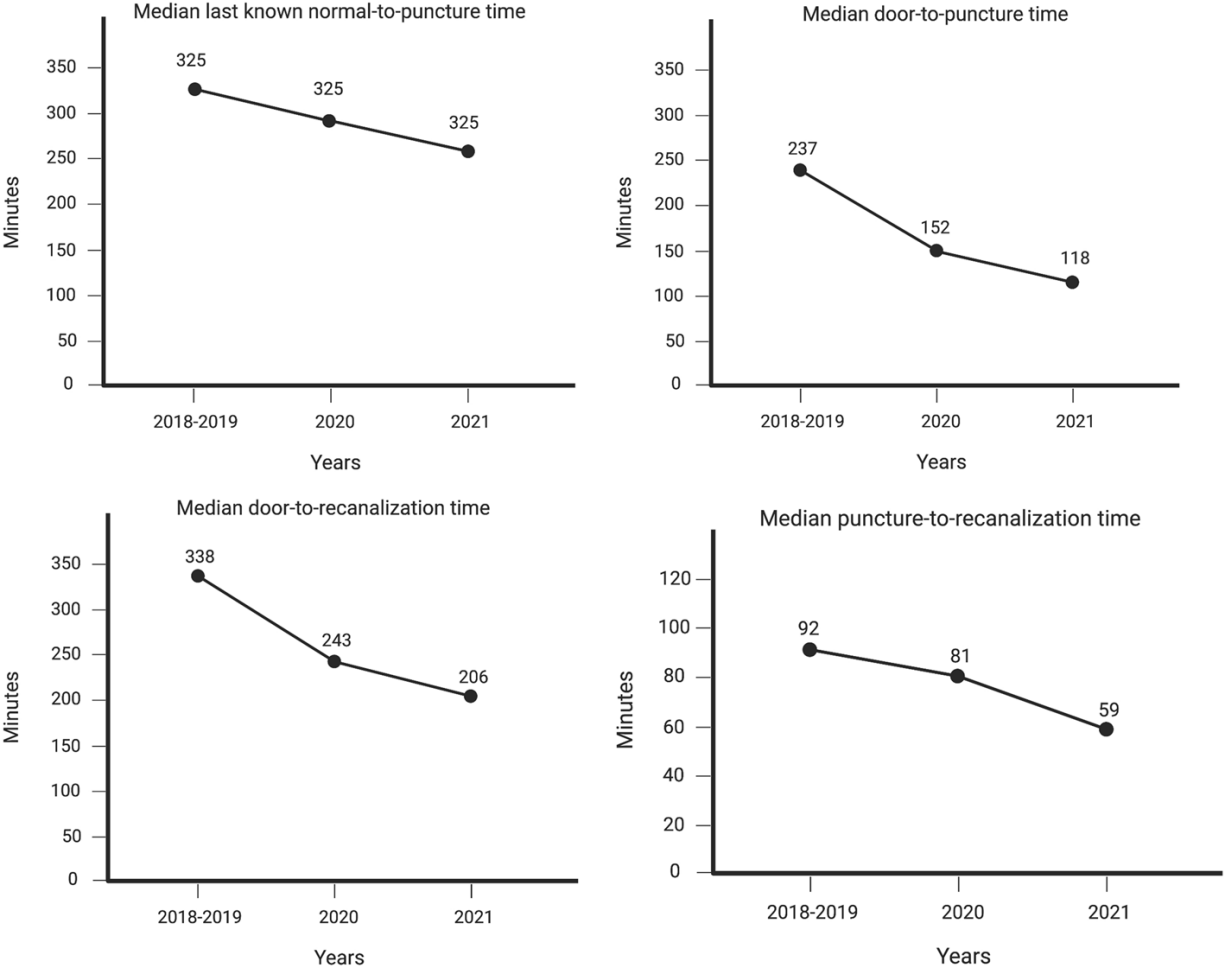
Median LKNPT, DPT, DRT, and PRT (min) from 2018 to 2021. All measurements showed a decreasing trend across the study period

Post-intervention measures, such as the median LKNPT was shorter post-intervention (255 vs. 325 min, *P*< 0.05; Table 3, Fig. 1). Similarly, DPT, DRT, and PRT were all significantly (*P* < 0.05) shorter in the post-intervention period versus pre-intervention period (118 vs. 237 min; 206 vs. 338 min; and PRT 59 vs. 92 min, respectively).

**Table 3:** Time metrics (min) for different phases of the study. P values are provided for each component. P1: *P* value for the pre-intervention vs Interim-intervention comparison. P2: *P* value for the pre-intervention vs post-intervention comparison. P3: *P* value for the interim-intervention vs post-intervention comparison

	Pre-intervention	Interim-intervention	post-intervention	P	P1	P2	P
LKNPT(IQR)	325.0 (301.0, 503.6)	291.0 (220.0, 540.0)	255.0 (186.5, 424.0)	0. 048	0.686	0.060	0.372
DPT(IQR)	237.0 (203.8, 298.0)	152.0 (105.0, 203.0)	118.0 (98.0, 153.5)	0.000	0.001	0.000	0.039
DRT(IQR)	338.0 (291.3, 407.3)	243.0 (177.0, 322.0)	206.0 (143.0, 238.0)	0.000	0.014	0.000	0.019
PRT(IQR)	92.0 (57.5, 125.3)	81.0 (52.0, 118.0)	59.0 (40.5, 91.0)	0.047	0.184	0.056	0.049

Abbreviations—*LKNPT* Last known normal-to-puncture time, *DPT* Door-to-puncture time, *DRT* Door-to-recanalization time, *PRT p*uncture-to-recanalization time

**Fig. 2:**
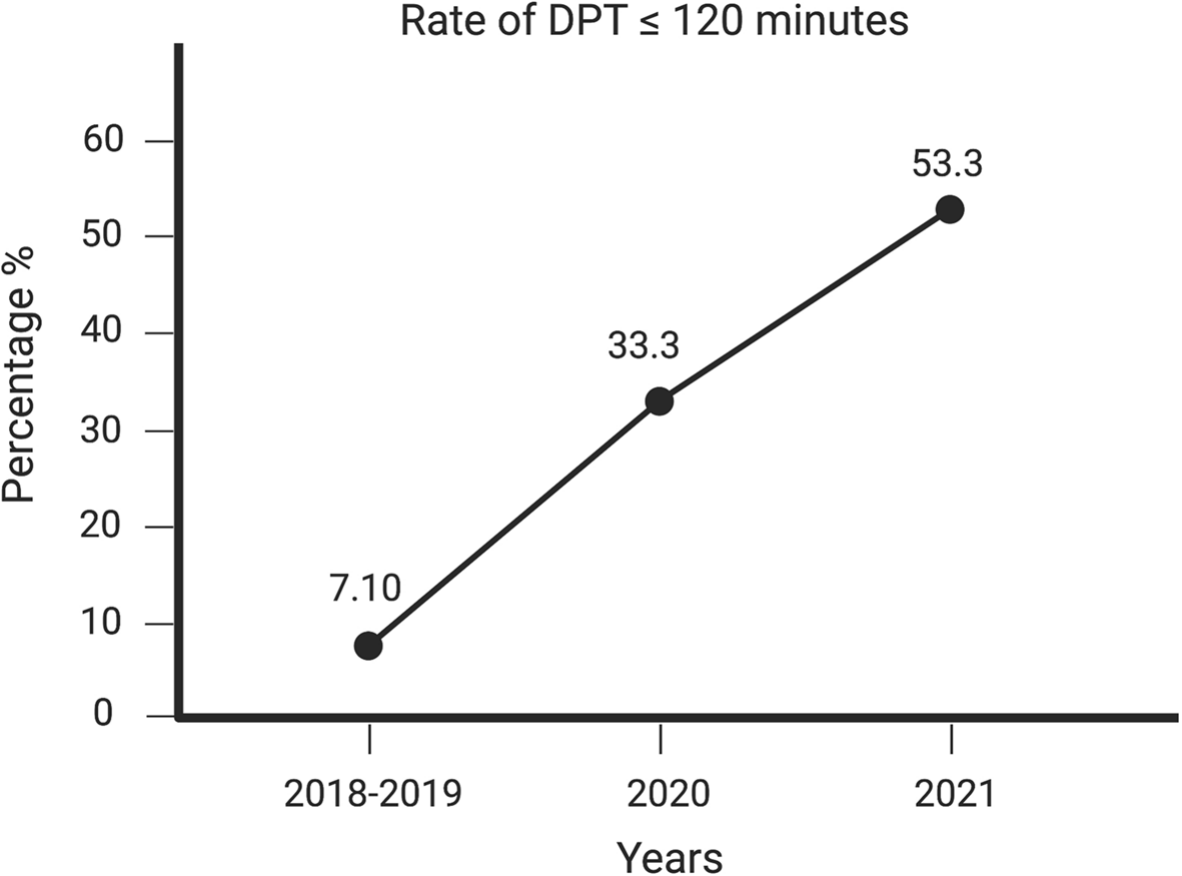
The target goal of DPT ≤ 120 min showed consistent improvements over the study period

The target goal of DPT ≤ 120 min is illustrated in Table 4 and Fig. 2. The target goal was statistically significant (*P* = 0.006) and showed consistent improvement.

**Table 4:** Target goal of DPT ≤ 120 min for different phases of the study. The P value is provided

Door-to-puncture time (min)	Pre-intervention	Interim-intervention	Post-intervention	*P* value
	*N* = 14	*N* = 39	*N* = 45	
DPT ≤ 120 min, n (%)	1 (7.1%)	13 (33.3%)	24 (53.3%)	0.006

The target goal of DPT ≤ 120 min improved from 7.1% in 2018–2019 to 33.3% in 2020, and 53.30% in 2021 in the post-intervention period (*P* = 0.006).

No statistically significant difference was observed concerning the rate of pneumonia, TICI post ≥ 2b, mRS at discharge, inpatient mortality or hospice discharge, and patient mortality at three months. In the post-intervention group, 55.6% had a favorable outcome, and only 21.4% had a favorable outcome measured at 3 months (*P* = 0.026) in the pre-intervention group (Table 5).

**Table 5:** Comparison of patient outcomes at different phases of the study. P1: *P* value for the pre-intervention vs Interim-intervention comparison. P2: *P* value for the pre-intervention vs post-intervention comparison. P3: *P* value for the interim-intervention vs post-intervention comparison

	Pre-intervention, *n* = 14	Interim-intervention, *n* = 39	Post-intervention, *n* = 45	P	P1	P2	P3
Pneumonia, n, %	6 (42.9%)	16 (41.0%)	15 (33.3%)	0.702	0.905	0.516	0.466
TICI post ≥ 2b, n, %	11 (78.6%)	34 (87.2%)	37 (82.2%)	0.709	0.440	0.759	0.531
Urinary tract infection, n, %	1 (9.1%)	0 (0.0%)	4 (8.9%)	0.169	0.092	0.838	0.056
sICH, n, %,	5 (35.7%)	6 (15.4%)	4 (8.9%)	0.052	0.108	0.015	0.359
mRS discharge (IQR)	4.0 (4.0,5.0)	4.0 (2.0,5.0)	3.0 (1.0,5.0)	0.515	0.976	0.516	0.486
Inpatient Mortality/hospice discharge, n, %	3 (21.4%)	9 (23.1%)	10 (22.2%)	0.991	0.899	0.095	0.926
The favorable outcome at 3 months, n, %	3 (21.4%)	16 (41.0%)	25 (55.6%)	0.067	0.190	0.026	0.184
Mortality at 3 months, n, %	7 (50.0%)	15 (38.5%)	13 (28.9%)	0.319	0.452	0.145	0.353

Ninety day outcomes according to the interval of DPT are summarized in Table 6, indicating a non-significant trend toward better outcomes among patients who achieved a DPT of 120 min or less, when compared to patients with a DPT of > 180 min.

**Table 6:** Outcome of different DPT (minutes). P1: *P* value for the pre-intervention vs Interim-intervention comparison. P2: *P* value for the pre-intervention vs post-intervention comparison. P3: *P* value for the interim-intervention vs post-intervention comparison

	DPT ≤ 120	120 < DPT ≤ 180	DPT > 180	P1	P2	P3
MRS (0–2), %	21 (55.3%)	12 (44.4%)	11 (33.3%)	0.390	0.064	0.379
MRS (3–6), %	17 (44.7%)	15 (55.6%)	22 (66.7%)	0.390	0.064	0.379

P1: DPT ≤ 120 vs 120 < DPT ≤ 180; P2: DPT ≤ 120 vs DPT > 180; P3: 120 < DPT ≤ 180 vs DPT > 180

## Discussion

Our findings in this study indicate that process optimization measures can successfully be implemented to shorten DPT, DRT, PRT, and LKNPT according to available hospital resources. We observed significant improvements in both arrivals to arterial puncture as well as the PRT during the study period. An increase in achieving a 90-day favorable outcome (mRS score of 0 to 2) was also observed, with favorable outcomes non-significantly more common among patients with shorter DPT, as has been shown in prior studies [3, 4].

The most recent American Stroke Association (ASA) guidelines recommends a goal for door-to-endovascular treatment time being restricted to within 120 min of stroke-onset [12]. Following the ASA recommendations, our center successfully improved the deadline of DPT ≤ 120 min from 7.1% in 2018–2019 to 33.3% in 2020 and 53.30% in the 2021 post-intervention period (*P* = 0.006). With every minute counting to manage such cases, a 90-min DPT for receiving endovascular treatment is considered for optimal management [13]. A recent study also demonstrated there were no significant differences in long-term thrombectomy outcomes among proximal anterior circulation patients who were selected based on non-contrast CT compared as compared to those selected with CTP or MRI in the extended window of 6 to 24 h [14]. Therefore, when possible, the patients may be selected without advanced or additional imaging beyond the CT in order to shorter DPT. Delays in stroke care and reperfusion treatment were a global challenge during the COVID-19 pandemic, corresponding to a global decline in the volume of stroke hospitalizations during the COVID-19 period [15–18]. The Society of Vascular and Interventional Neurology (SVIN) provided a formal guidance statement for recalibrating stroke workflow to protect frontline healthcare workers, their families and colleagues, with individualization of stroke treatment according to patient needs during the COVID-19 pandemic [19].

Strategies to optimize DPT, DRT, PRT, and LKNPT are critical to improve patient outcomes. Once acute stroke patients arrive at the hospital, resources must be allocated to rapidly identify patients with suspected ischemic stroke and intracranial occlusion, and mobilize personnel in order to treat using endovascular interventions. These processes necessitate the involvement of pre-hospital transfer services alongside ED personnel, neurologists, nurses, radiologists, interventionalists, and the hospital administration department. Only by involving each of these stakeholders can the most effective treatment be provided in the timeliest manner.

Our study has some limitations. The study is a retrospective study in a single hospital with a small data sample size. Prospective multicenter studies and larger sample data sizes are required to analyze shortened DRT and patient outcomes. Despite these limitations, we believe accomplishments at our center can provide a framework for other stroke centers to improve their patient outcomes.

## Conclusions

This study demonstrated that multidisciplinary cooperation could shorten the time to endovascular treatment, with the potential to improve long-term patient outcomes. We call on stroke centers and healthcare providers to internally review their local paradigms to evaluate where improvements can be made to safely expedite care.

## Data Availability

The datasets generated and analyzed during the current study are not publicly available due to the non-disclosure agreement in institutional review board restrictions. Availability of data and materials were available by contacting corresponding authors on reasonable request.

## Abbreviations

AF: Atrial fibrillation
ASPECTS: Alberta stroke program early CT score
ASA: American stroke association
CAD: Coronary heart disease
CE: Cardioembolism
CKD: Chronic kidney disease
DM: Diabetes mellitus
ED: Emergency department
EVT: Endovascular therapy
DNT: Door-to-needle time
DPT: Door-to-puncture
DRT: Door-to-recanalization time
ICA: Internal carotid artery
IQR: Interquartile range
IV: Intravenous
LAA: Large-artery atherosclerosis
LKNPT: Last known normal time to-puncture time
mRS: Modified Rankin Scale
mTICI: Modified treatment in cerebral infarction
NIHSS: National Institute of Health Stroke Scale
PRT: Puncture-to-recanalization time
SD: Standard deviations
sICH: Symptomatic intracranial hemorrhage
SOE: Stroke of undetermined etiology
STROBE: STrengthening the Reporting of OBservational Studies in Epidemiology
SUE: Stroke of undetermined etiology
SVIN: Society of Vascular and Interventional Neurology
SVO: Small vessel occlusion
TOAST: Trial of ORG 10,172 in acute stroke treatment
